# Use of the Kalman Filter for Aortic Pressure Waveform Noise Reduction

**DOI:** 10.1155/2017/6975085

**Published:** 2017-05-22

**Authors:** Frank Lam, Hsiang-Wei Lu, Chung-Che Wu, Zekeriya Aliyazicioglu, James S. Kang

**Affiliations:** ^1^Department of Electrical & Computer Engineering, California State Polytechnic University, Pomona, 3801 W. Temple Ave, Pomona, CA 91768, USA; ^2^Keck Graduate Institute of Applied Life Sciences, 535 Watson Dr, Claremont, CA 91711, USA; ^3^Taipei Medical University Hospital, Taipei Medical University, 252 Wuxing St, Xinyi District, Taipei City, Taiwan

## Abstract

Clinical applications that require extraction and interpretation of physiological signals or waveforms are susceptible to corruption by noise or artifacts. Real-time hemodynamic monitoring systems are important for clinicians to assess the hemodynamic stability of surgical or intensive care patients by interpreting hemodynamic parameters generated by an analysis of aortic blood pressure (ABP) waveform measurements. Since hemodynamic parameter estimation algorithms often detect events and features from measured ABP waveforms to generate hemodynamic parameters, noise and artifacts integrated into ABP waveforms can severely distort the interpretation of hemodynamic parameters by hemodynamic algorithms. In this article, we propose the use of the Kalman filter and the 4-element Windkessel model with static parameters, arterial compliance *C*, peripheral resistance *R*, aortic impedance *r*, and the inertia of blood *L*, to represent aortic circulation for generating accurate estimations of ABP waveforms through noise and artifact reduction. Results show the Kalman filter could very effectively eliminate noise and generate a good estimation from the noisy ABP waveform based on the past state history. The power spectrum of the measured ABP waveform and the synthesized ABP waveform shows two similar harmonic frequencies.

## 1. Introduction

### 1.1. Motivation

According to Asgari et al., any type of clinical application that requires the extraction and interpretation of physiological signals or waveforms is susceptible to corruption by noise or artifacts [1]. Real-time hemodynamic monitoring systems are important for clinicians to assess the hemodynamic stability of surgical or intensive care patients by interpreting hemodynamic parameters generated by an analysis of aortic blood pressure (ABP) waveform measurements. Hemodynamic monitoring instruments can measure ABP waveforms invasively using catheters that are inserted into the arteries [2] or utilize noninvasive sensors that obtain arterial pressure pulses without catheter insertion. Regardless of the method used for acquiring ABP waveforms, ABP waveforms can be used as an input into hemodynamic parameter estimation algorithms to estimate hemodynamic parameters such as cardiac output (CO) or stroke volume (SV) [2, 3]. Since hemodynamic parameter estimation algorithms often have to detect events and features from measured ABP waveforms in order to generate hemodynamic parameters, the integration of noise and artifacts into ABP waveforms can severely distort the interpretation of hemodynamic parameters by hemodynamic algorithms.

There are many possible sources of noise and artifacts in real-time hemodynamic monitoring systems. Such sources can arise from the instrument or sensors unintentionally picking up environmental noise, lack of channel synchronization or delay between instruments that are used in conjunction with each other, arterial line containing partial or complete blockage that causes signal damping, patient's physical movements or physiological abnormalities, and respiratory modulation due to ventilator use on patients.

Lawless has shown through a study in the pediatric ICU that the majority of alarms sounded by clinical equipment were actually not clinically important—which facilitates the need for improvements to be made in patient care monitoring systems [4]. One method for improving patient monitoring systems revolves around noise and artifact reduction. There have been many past attempts to reduce noise and artifacts of physiological ABP waveforms for use in patient monitoring systems. Asgari et al. have shown the capabilities of noise reduction by recognizing valid and invalid cardiac cycles—in an attempt to avoid integrating noise and artifacts [1], while Li et al. have shown the feasibility of avoiding errors caused by noise, artifacts, or missing portions of data when performing an estimation of heart rate (HR)—using signal quality indices in conjunction with the Kalman filter [5]. While the researches of Asgari et al. and Li et al. have provided valid insights in preventing noise integration, we propose the use of the Kalman filter, which is an adaptive filter, and the 4-element Windkessel model of arterial circulation with static parameters to both generate and reduce noise from ABP waveforms.

### 1.2. Kalman Filter

**Figure 1:**
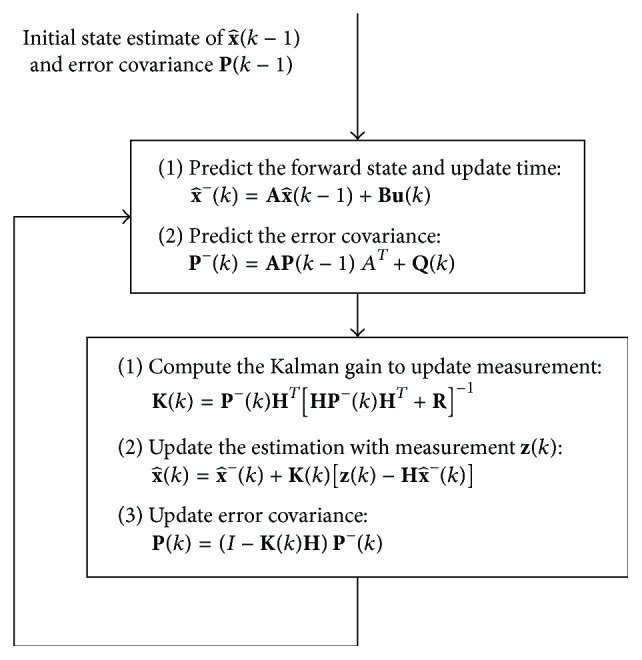
Complete system level diagram depicting the recursive operations of the Kalman filter, adapted from Welch and Bishop.

The Kalman filter is a recursive optimal estimator algorithm that utilizes parameters derived from indirect, inaccurate, and uncertain observations. From the uncertainty, the Kalman filter can provide a recursive solution that minimizes the mean square error of the estimated parameters—allowing for the state of a dynamic deterministic system to predict the future outputs of the deterministic system based on the system's past [6]. The recursive steps of the Kalman filter algorithm to yield the best estimate of **x**(*k*) are depicted in Figure 1.

The state equation of the Kalman filter, modeling the transformation of the process state, is written as

(1) $$\begin{array}{c} x\left(\;k+1\;\right)=Ax\left(\;k\;\right)+Bu\left(\;k\;\right)+w\left(\;k\;\right), \end{array}$$

where

 
**x**(*k*) is the *n* × 1 system vector representing the state of the dynamic deterministic system,
 
**A** is the *n* × *n* state system matrix,
 
**B** is the *n* × *m* input matrix,
 
**u**(*k*) is the *m* × 1 known input signal,
 
**w**(*k*) is the process white noise [6].

The measurement equation describing the relationship between the process state and the measurements is written as

(2) $$\begin{array}{c} z\left(\;k\;\right)=Hx\left(\;k\;\right)+v\left(\;k\;\right), \end{array}$$

where

 
**z**(*k*) is the known output measurement signal,
 
**H** is the *m* × *n* output matrix,
 
**v**(*k*) is the process measurement noise.

Based on the definitions of (1) and (2), the random variables **w**(*k*) and **v**(*k*) are independent representations of the process and measurement noises—each carrying different Gaussian probability density functions representing zero mean white noise. The covariance matrix of the process noise, **w**(*k*), is **Q**(*k*), also known as the process noise covariance. Conversely, the covariance matrix of the measurement noise, **v**(*k*), is **R**(*k*), also known as the measurement noise covariance [7].

In addition to the variable definitions for the state equation and measurement equation of the Kalman filter, both **Q**(*k*) and **R**(*k*) were chosen to be constants,

where

 
**Q**(*k*) is the *n* × *n* process noise covariance,
 
**R**(*k*) is the *m* × *m* measurement noise covariance,
 
**P**^−^(*k*) is the *n* × *n* a priori estimate error covariance,
 
**P**(*k*) is the *n* × *n* a posteriori estimate error covariance,
 
**K**(*k*) is the *n* × *m* Kalman gain.

In the prediction step of the Kalman filter, the a priori estimate state ${\hat{x}}^{-}(k)$ is estimated based on previous states $\hat{x}(k)$ and **A**. To gauge the deviation of the model's output and the measurements, the a priori estimate produces an estimate of its predicted measurement, $\hat{x}(k)=H{\hat{x}}^{-}(k)$, which is subtracted from the actual measurement **z**(*k*) in order to obtain a residual $z(k)-H{\hat{x}}^{-}(k)$. The a priori estimate error **e**^−^(*k*) is defined as $x(k)-{\hat{x}}^{-}(k)$ while the a posteriori estimate error **e**(*k*) is defined as $x(k)-\hat{x}(k)$.

The Kalman gain **K**(*k*) is calculated as the blending factor that minimizes the a posteriori error covariance. If the a priori error **e**^−^(*k*) is small, then **K**(*k*) would also be small—resulting in a minimal amount of correction to be had in $K(k)[z(k)-H{\hat{x}}^{-}(k)]$. When it is determined that the error of the a priori estimate is small, it is inferred that there is no need for correction, and the system model is trusted over the measurements. Conversely, if the a priori error **e**^−^(*k*) is big, then the a posteriori estimate of the states $\hat{x}(k)$ will depend more on the measurement **z**(*k*), rather than the a priori estimate ${\hat{x}}^{-}(k)$ [7].

## 2. Methods

### 2.1. Windkessel System Model

To implement the Kalman filter, a model to represent arterial circulation is necessary for the process state. The Windkessel model of arterial circulation is a physiological lumped parameter model based on the modeling of the arterial system as an air reservoir and was chosen because of its low computational costs—as a result of its simplification of both the fluid dynamics of the blood and the mechanical dynamics of the arterial blood vessels into lumped parameters. Despite its simplicity, the Windkessel models have been shown to realistically mimic systemic arterial load because each of the independent parameters is based on real physiology [8]. Campbell et al. performed a study to best fit pressure waveforms using aortic flow and the 3-element Windkessel model—determining that there is an agreement between the model's prediction ability and experimental data [9]. Compared to the 3-element Windkessel model, the 4-element Windkessel model used in this study contains four parameters that model arterial circulation: arterial compliance *C*, peripheral resistance *R*, aortic impedance *r*, and the inertia of blood *L* [10].

**Figure 2:**
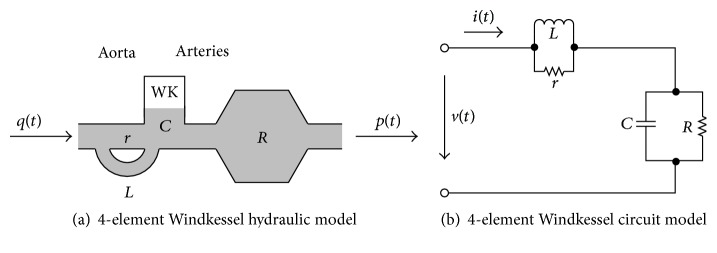
Different Windkessel model diagrams, adapted from Westerhof et al.

Because of the close relationship between mechanical and electrical systems, the Windkessel model can be represented as either a mechanical or electrical system—which allows for a source of time varying flow or current to be used as an input to generate a source of time varying pressure or voltage, respectively.  Figure 2 shows the 4-element Windkessel model analogs—both of which are capable of generating ABP waveforms by modeling the propagation of blood as it interacts with the aorta, the arterial tree, or both the aorta and the arterial tree—depending on the Winkessel model used [10].

In order to model the process state and test the 4-element Windkessel model's capabilities, linear values of the Windkessel parameters had to be determined to best represent the pig data described in Section 2.2. Segers et al. performed an experiment to estimate the 4-element Windkessel model parameters with the use of both flow and pressure data measured from a pig [11]. The linear Windkessel parameters estimated by Segers et al. shown in Table 1 were used as the static parameters for this article.

#### 2.1.1. Windkessel Model Differential Equations

The electrical analog of the 4-element Windkessel model shown in Figure 2(b) can be mathematically described by three differential equations where

the differential equation representing the parallel resistor and inductor is
  (3) $$\begin{array}{c} \frac{r}{L}i\left(\;t\;\right)-\frac{r}{L}i_{L}\left(\;t\;\right)=\frac{di_{L}\left(t\right)}{dt}, \end{array}$$
the differential equation representing the parallel resistor and capacitor is
  (4) $$\begin{array}{c} \frac{1}{C}i\left(\;t\;\right)-\frac{1}{RC}v_{C}\left(\;t\;\right)=\frac{dv_{C}\left(t\right)}{dt}, \end{array}$$
the differential equation representing the series impedance of the circuit is
  (5) $$\begin{array}{c} v\left(\;t\;\right)=i\left(\;t\;\right)r-i_{L}\left(\;t\;\right)r+v_{C}\left(\;t\;\right). \end{array}$$

#### 2.1.2. Windkessel Model Discretization

The Kalman filter can be implemented as a digital filter with the 4-element Windkessel as the system model. Because digital filters perform computations in discrete time, the difference equations that represent the 4-element Windkessel can be derived using the Euler forward method as

(6) TsrLik−TsrL+1iLk=iLk+1,Ts1Cik−Ts1RC+1vCk=vCk+1,vk=ikr−iLkr+vCk,

where *T*_*s*_ refers to the sampling period that was chosen based on the 500 Hz sampling rate of the pig data [12].

From the difference equations, the coefficient matrices representing the 4-element Windkessel can be written as

(7) A=−1RCTs+100−rLTs+1,B=1CTsrLTs,

and the coefficient matrices representing the output can be expressed as

(8) H=1−r,Q=r.

### 2.2. Data Sources

**Figure 3:**
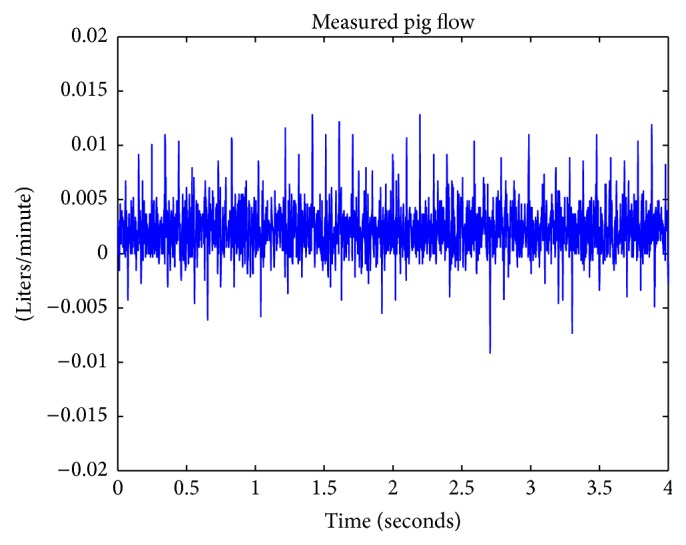
Depiction of aortic flow measured from a pig.

**Figure 4:**
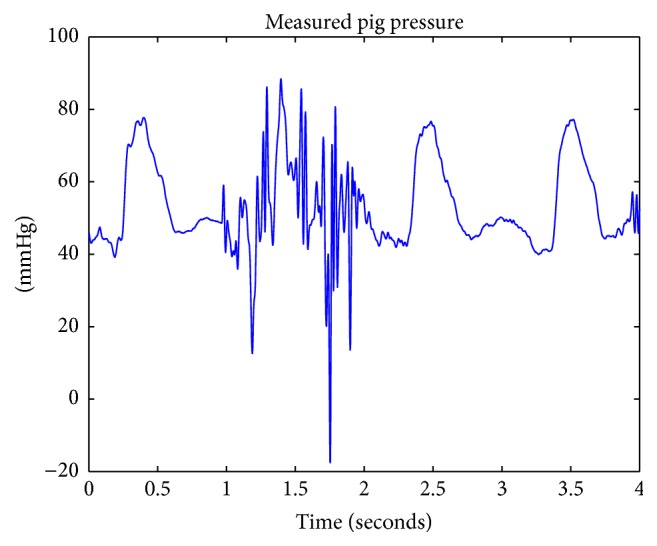
Depiction of aortic pressure measured from a pig.

In order to simulate and generate synthetic ABP waveforms, pig data was acquired from a previous study by Cannesson et al. [12]. In that study, healthy pigs with a normal anatomy were sutured with many lines to measure different circulatory system waveforms. The pig's aortic flow was obtained from measurements with an aortic flow probe—in a nine-hour open chest procedure at the aortic root of a healthy 87-kilogram female pig. Aortic blood pressure was measured with the implementation of a catheter at the aortic root. Prior to opening the chest, gas anesthesia and isoflurane were administered in order to prepare the pig for the procedure. Local block lidocaine 1–3 mg/kg was administered through use of an IV drip—in order to provide continuous amounts of sedative to the area of open incision. The animal was euthanized at the end of the study. The measured pig flow data is depicted in Figure 3 and was used as an input into the 4-element Windkessel. The measured output pressure data is shown in Figure 4 and represents the expected output of the 4-element Windkessel. In Figure 4, noise and artifacts were recorded between 1 and 2 seconds.

## 3. Results and Discussion

In order to reduce noise and artifacts from ABP waveforms, this simulation incorporates the Kalman filter with the 4-element Windkessel model representing the pathway of blood as it circulates through the aorta and arteries. Measured pig flow data was used as an input into the system in order to synthesize an ABP waveform, while measured pig aortic pressure data was used as a baseline output for comparison.  Figure 6 depicts the measured pig ABP waveform superimposed with the Kalman filter's estimation. In comparison with conventional methods of noise reduction which entails the elimination of data that exists at lower and higher bound frequencies with high-pass or low-pass filters—since the Kalman filter operates through the prediction of future states based on prior state knowledge, it is able to strategically eliminate noise and estimate an ABP waveform containing low amounts of noise without the absolute loss of high or low frequency waveform data.

**Figure 5:**
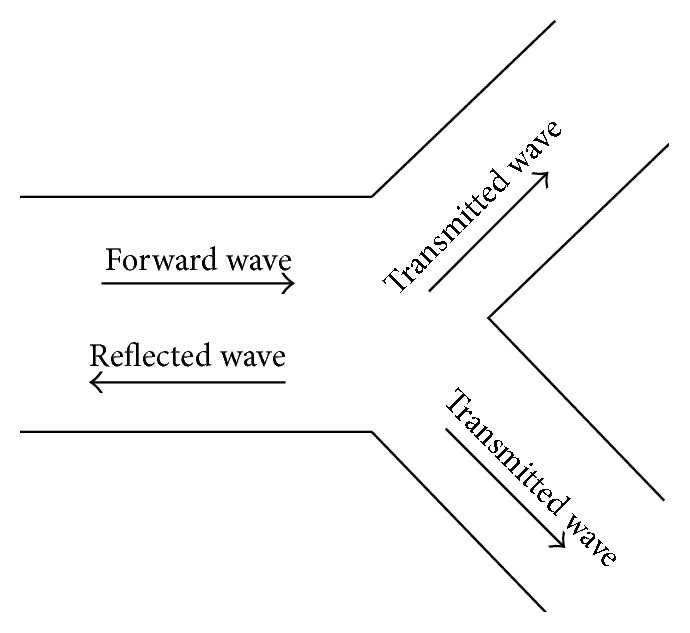
Depiction of the forward and reflected waves at arterial bifurcations, adapted from Duan et al.

**Figure 6:**
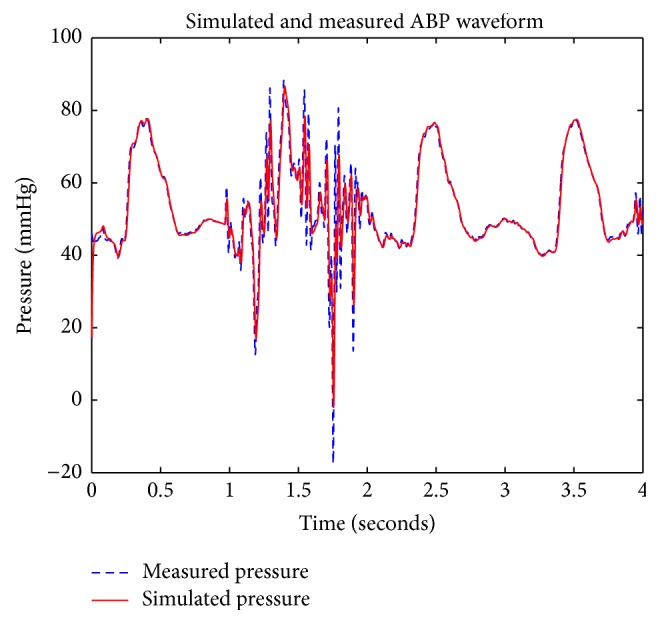
Kalman filter output of aortic pressure.

**Figure 7:**
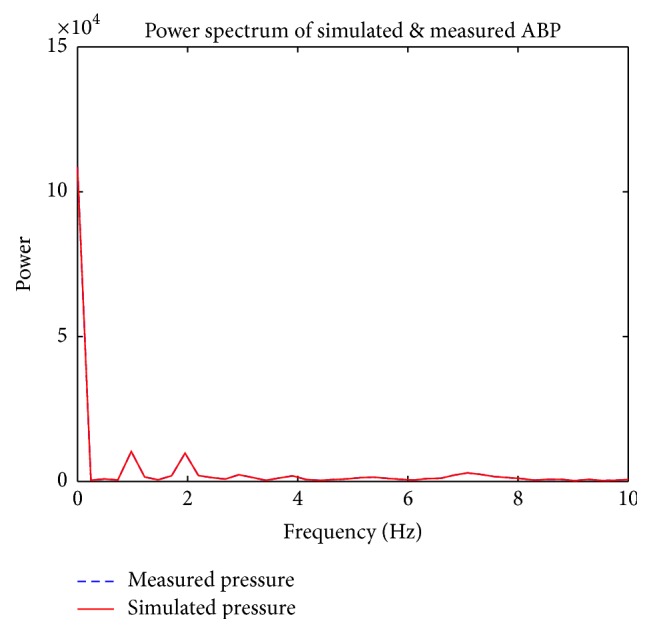
Power spectrum of arterial pressure.

Figure 7 is a superimposed depiction of the power spectrum corresponding to both the measured pig ABP waveform and the synthesized ABP waveform generated by the Kalman filter, which indicates that the frequency distribution of both the measured and the generated waveform is very similar. According to Pittman et al., ABP waveforms are generated as a result of the summation of a forward wave, which is generated by the ejection of blood at the left ventricle in the forward direction, and reflected waves resulting from blood flowing in the reverse direction at arterial bifurcations [13]. An example of an arterial bifurcation is depicted in Figure 5. By recording arterial pulse tracings, Hoeksel et al. determined that, in blood pressure waveforms, the amplitude of the ABP waveform during the onset of blood ejection (systole) can be represented as the forward wave, and the duration of blood ejection (diastole) can be represented as a percentage of the amplitude of the systolic portion of the ABP waveform due to wave reflections at arterial bifurcations [14, 15]. Duan et al. experimentally studied the transmission and reflection characteristics of blood pulse waveforms in dogs and mathematically determined that the amplitudes of wave reflections can be as high as 20% of the forward wave's amplitude [16]. Because the forward and reflected waves of ABP waveforms are separated by the dicrotic notch, there are two expected harmonic peaks that should exist when ABP waveforms are analyzed using Fourier analysis. Figure 7 shows two harmonic peaks, where the first harmonic at ≈ 1 Hz represents the forward wave and the second harmonic at ≈ 2 Hz represents the reflected wave. The DC component found at 0 Hz can be ignored because it represents the average of all the samples used, a harmonic that does not pertain to the natural characteristics of the aortic tree. Changes are more visible in the time domain, as the amplitude of the original measured pressure is changed, but in the frequency domain the corresponding harmonics remain the same. These results demonstrate that the waveform estimated by the Kalman filter is realistic and is comparable to real ABP waveforms that are measured from a pig.

**Figure 8:**
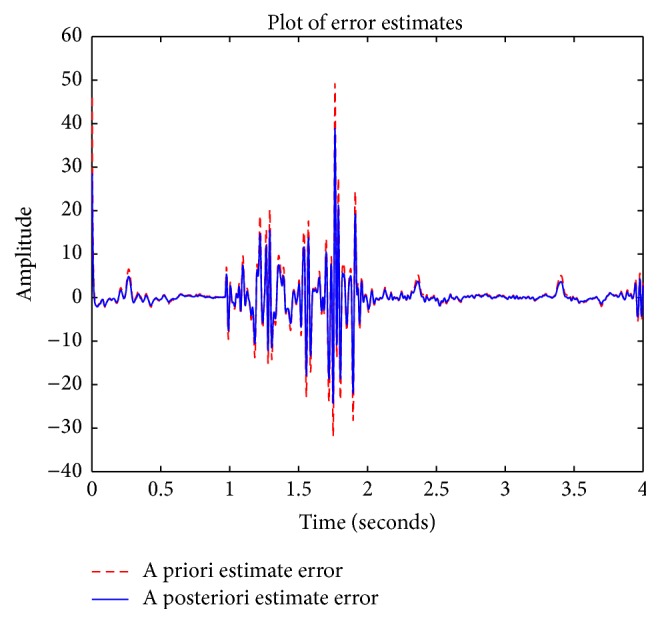
Kalman filter estimation error.

A limitation of the Kalman filter's ability to reduce noise stems from the accumulation of errors with each completed iteration. As a result, the Kalman filter's ability to reduce noise from ABP waveforms becomes hindered with time. In order to gauge how such error accumulation can impact the Kalman filter's performance, a comparison of estimation errors between the measured pig ABP waveform and the synthesized ABP waveform generated by the Kalman filter can be made by subtracting the measured pig ABP waveform and the synthesized ABP waveform generated by the Kalman filter. A plot of estimation error is shown in Figure 8, where it can be seen that there is a negligible increase in estimation error when comparing the initial estimation at 0 seconds with that at 2 seconds.

**Figure 9:**
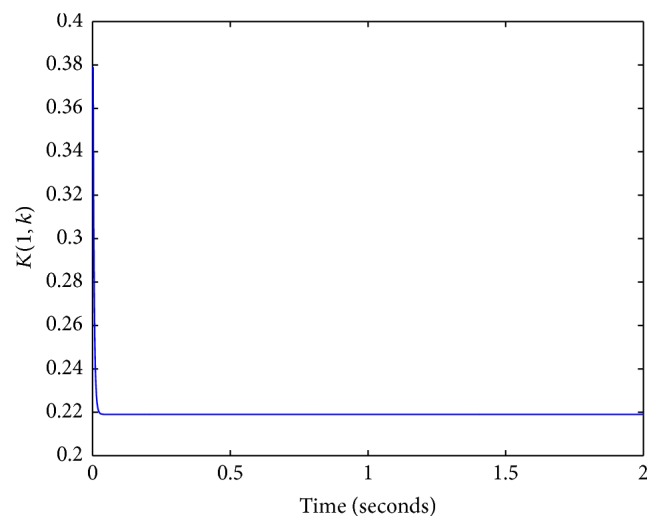
Kalman gain.

In order to generate the best possible output, the Kalman filter incorporates the measured pig ABP waveform in its computation. As a result, the final estimate of a state is a weighted combination of the state predicted by the system model and the state estimated from the output observed. The weighing between the model and the measurement is determined by the Kalman gain matrix where if measurements are noisy, then model prediction is weighted more heavily, but if measurements have lower amounts of noise, then measurements are weighted more heavily. Even though Windkessel parameters can vary in a wide range from one subject compared to another, the weighing process performed by the Kalman gain adjusts and compensates between measurements and the model in order to allow for the use of static linear Windkessel parameters estimated by Segers et al. shown on Table 1 to generate a very accurate estimation of ABP waveforms. Values of the Kalman gain that are closer to 0 indicate that the model was preferred over the measurements. However, values of the Kalman gain that are closer to 1 indicate that the measurements were preferred over the model. The Kalman gain is a function of the certainty of the measurements and the current state estimate. At steady state, Figure 9 shows the Kalman gain to be ≈ 0.22—which indicates that the model was preferred more over the measurements and also infers that the 4-element Windkessel model using static Windkessel parameters is a good representative model for aortic circulation.

## 4. Conclusion

In this article, we proposed the use of the Kalman filter and the 4-element Windkessel model to represent aortic circulation for generating accurate estimations of ABP waveforms by reducing noise and artifacts. In our simulation, the Kalman filter was shown to very effectively eliminate noise and generate a good estimation from the noisy ABP waveform. In order to confirm the realism of the Kalman filter's estimation, the frequency spectrum of the synthesized ABP waveform was observed and found to contain both a forward frequency and reverse frequency—both of which are characteristics that are observed in real-life ABP waveforms. In addition, the similarity of the frequency spectrum of the synthesized ABP waveform compared to the frequency spectrum of the measured pig ABP waveform also demonstrates the realism of the waveform generated by the Kalman filter.

## Figures and Tables

**Table 1:** Linear Windkessel model parameters, adapted from Segers et al.

Windkessel parameter	Systemic circulation parameter
*R*	1.72 mmHg/(ml/s)
*C*	0.48 ml/mmHg
*r*	0.105 mmHg/(ml/s)
*L*	0.0059 mmHg/(ml/s^2^)
